# Barriers and facilitators for intensive care nurses in using clinical decision support systems for intravenous Y-site compatibility in intensive care: A survey study

**DOI:** 10.1016/j.ijnsa.2026.100637

**Published:** 2026-07-20

**Authors:** Annabel Peyravi Latif, Alessio Degl’Innocenti, Bengt Nellgård, Axel Wolf

**Affiliations:** a Department of Anaesthesiology and Intensive Care Medicine, Institute of Clinical Sciences, Sahlgrenska Academy, University of Gothenburg, Gothenburg, Sweden; b Regionhälsan, Region of Västra Götaland, Gothenburg, Sweden; c Department of Anaesthesiology and Intensive Care Medicine, Institute of Clinical Sciences, Sahlgrenska University Hospital, Mölndal, Sweden; d Centre for Ethics, Law and Mental Health (CELAM), Department of Psychiatry and Neurochemistry, Institute of Neuroscience and Physiology, Sahlgrenska Academy, University of Gothenburg, Gothenburg, Sweden; e University of Gothenburg Centre for Person-Centred Care (GPCC), Sahlgrenska Academy, University of Gothenburg, Sweden; f Department of Anaesthesiology and Intensive Care Medicine, Sahlgrenska University Hospital/Östra, Gothenburg, Region of Västra Götaland, Sweden; g Institute of Nursing and Health Promotion, Oslo Metropolitan University, Oslo, Norway; h Institute of Health and Care Sciences, Sahlgrenska Academy, University of Gothenburg, Gothenburg, Sweden

**Keywords:** Clinical decision support systems, Intensive care nurses, Intensive care unit, Intravenous drug administration, Intravenous drug compatibility, Y-site drug compatibility

## Abstract

**Background:**

Patients in intensive care units frequently receive multiple intravenous medications, increasing the risk of medication errors, including incompatible Y-site drug co-administration. Drug incompatibility can lead to serious clinical consequences, yet evidence supporting the safety of many commonly co-administered drug combinations is limited. Clinical decision support systems may help reduce medication errors and improve patient care, but challenges remain in their design and adaptation to complex and dynamic clinical settings such as the intensive care unit.

The Swedish Y-site compatibility system was developed by pharmacists and, it is a website that enables users to assess potential Y-site compatibility between intravenously administered drug pairs. However, the website was not specifically designed for intensive care setting, where the need for this information is greatest.

**Objective:**

To identify barriers and facilitators influencing intensive care nurses’ use of clinical decision support systems to enhance the safety of drug administration, with a focus on the Swedish Y-site Compatibility System.

**Methods:**

A digital survey, inspired by the BEhavior and Acceptance fRamework (BEAR), was distributed to all 296 intensive care unit nurses in clinical service at an intensive care unit at a large University Hospital in Sweden. The survey included respondent characteristics, intravenous drug administration practices, general perceptions of clinical decision support systems, and attitudes towards the Swedish Y-site compatibility system.

**Results:**

A total of 126 intensive care unit nurses (43%) responded to the survey. Among them, 67% reported administering intravenous medications without knowing if they are compatible when co-administered intravenously, and 18% reported performing such administration at least weekly. Most respondents (89%) used the Swedish Y-site compatibility system, and 82% would recommend it to others. No apparent barrier was identified among the surveys areas; social factors, clinical context, and implementation by assessing nurses’ perceptions, intravenous drug administration practices, and specific feedback on the Swedish Y-site compatibility system. Identified facilitators included the perceived value of clinical decision support systems in improving intravenous medication administration and patient care.

**Conclusions:**

Intensive care unit nurses reported positive views regarding clinical decision support systems, and the Swedish Y-site compatibility system was regarded as a valuable tool in supporting safer intravenous medication administration. Despite widespread use, many nurses still administer intravenous drugs without knowing its compatibility, indicating ongoing risk and a need for targeted education, and further development of the Swedish Y-site compatibility system to tailor it to the intensive care unit setting.

**Registration:**

Not registered.

**What is already known**

• Patients in intensive care units frequently receive multiple intravenous medications, increasing the risk of medication errors, including incompatible Y-site co-administration.
• Evidence supporting the safety of many commonly co-administered intravenous drug combinations is limited, contributing to uncertainty in clinical practice.
• Clinical decision support systems can reduce medication errors, but their effectiveness in intensive care settings depends on usability, implementation, and adaptation to complex clinical environments.

**What this paper adds**

• The clinical decision support system, the Swedish Y-site compatibility system, is widely used and valued by intensive care unit nurses to support safer intravenous medication administration.
• Despite high system use, many nurses report administering medications without knowing their compatibility, indicating persistent safety risks, along with uncertainty and work-related strain for intensive care nurses.
• Combining an intensive care unit–tailored clinical decision support system with ongoing education may reduce medication errors and enhance patient safety in intensive care settings.

Alt-text: Unlabelled box dummy alt text

## 1 Background

Patients in the intensive care unit often have multiple critical conditions, as well as underlying co-morbidities. This results in complex pharmaceutical treatment strategies of multiple intravenous medications (McDowell et al., 2010; Lyons et al., 2018). However, drugs are also a source for potential harm, as medication errors in hospital are a well-known patient safety issue (Preventing Medication Errors: Quality Chasm Series [Internet] 2007; Hodkinson et al., 2020; Kane-Gill et al., 2017). One notable safety concern in the intensive care unit is the simultaneous administration of incompatible medications through the same intravenous access line, commonly referred to as Y-site drug administration or co-administration (Trissel, 2026; Kanji et al., 2010; Taxis, 2003; Taxis and Barber, 2004).

Drug incompatibilities may result in physical or chemical changes, such as precipitation, colour alteration, gas formation, or loss of medication potency (Newton, 2009). This may lead to occlusion of intravenous lines, infusion pump malfunction, or organ damage and emboli (Nemec et al., 2008; Maki et al., 2006; McGee, 2003; Merrer et al., 2001; Benlabed et al., 2019; Bradley et al., 2026). A multi-centre, cross-sectional observational study found that 46% of the patients were receiving two or more medication infusions simultaneously as a Y-site drug administration. There were 40 incidents of inappropriate co-administration identified in 37 patients, representing a prevalence of 8.5% overall and 18.7% among patients receiving multiple infusions (Kanji et al., 2013). About 50–66% of commonly co-administered intravenous drug combinations used in adult intensive care units have no published evidence to supporting their safe administration (Kanji et al., 2010; D’Huart et al., 2019; Castells Lao et al., 2020).

The aim of clinical decision support systems is to deliver timely, evidence-based information that helps clinicians make informed choices regarding patient care. Clinical decision support systems can help reduce medication errors (Kawamoto et al., 2005; Kwan et al., 2020; Syrowatka et al., 2025; Syrowatka et al., 2023; Colin et al., 2025) enhance care transitions, increase efficiency, improve the effectiveness of healthcare services, and contribute to reducing overall costs (Sutton et al., 2020), as well as improve experienced cognitive load among intensive care unit nurses (Zhang et al., 2025). Despite the potential benefits of clinical decision support systems, challenges remain in the implementation and optimization of these systems in clinical practice (Phansalkar et al., 2013). Issues such as data accuracy, system interoperability, user interface design, and clinician acceptance and engagement must be carefully addressed to ensure the effectiveness and usability of clinical decision support systems in supporting safe and efficient intravenous therapy (Rommers et al., 2011; Harinstein et al., 2012; Eppenga et al., 2012; Camacho et al., 2020; Jansson et al., 2022).

A web-based clinical decision support systems, the Swedish Y-site compatibility system (Start - Blandbarhet [Internet] 2024), was developed in 2016 by pharmacists and introduced as a resource for Y-site drug compatibility information. The user can input intravenously administered drugs to retrieve information on potential Y-site compatibility between two drugs at a time. In 2022 the Swedish Y-site compatibility system was implemented as a nationwide clinical decision support system in Sweden. This open-access, evidence-based, pharmacist-reviewed clinical decision support system is accessible from both computers and mobile devices, such as smartphones or tablets. It provides clinically relevant Y-site drug compatibility information for healthcare professionals. What sets the system apart is its focus on drug compatibility at clinically used concentrations, incorporating pharmacists’ assessments of two-drug combinations. These assessments are based on peer-reviewed literature, pharmaceutical industry data, and expert pharmaceutical knowledge. However, the Swedish Y-site compatibility system was not specifically designed for the intensive care unit, where Y-site compatibility issues and co-administration are most prevalent.

Tailoring a clinical decision support system for the intensive care unit environment is recommended in literature, with emphasis on clinical relevance (Bakker et al., 2024; Wong et al., 2023). To our knowledge, such a clinical decision support system is not yet developed (Amaral and Cuthbertson, 2024). Therefore, the aim of this study is to identify relevant factors when designing a clinical decision support system in the intensive care unit, with regards of intravenous drug administration and especially Y-site drug compatibility (Westerbeek et al., 2021).

## 2 Method

### 2.1 Study setting and implementation context

The study was conducted at a large university hospital within a regional healthcare organisation in Sweden. The hospital includes several intensive care units providing care for critically ill patients across different specialties. The clinical decision support system evaluated in this study was originally implemented simultaneously across the entire regional healthcare organisation.

### 2.2 Respondents

A total of 296 intensive care unit nurses employed across six intensive care unit units at the university hospital’s three sites were employed and potentially eligible for inclusion in the study. Exclusion criteria included declining to have their answers used for research. Personnel on sick leave or parental leave were also sent the survey and are considered part of the eligible participant pool.

### 2.3 Procedure and questionnaire

The survey was designed by using the BEAR (BEhavior and Acceptance fRamework) (Camacho et al., 2020) which guided the selection of domains relevant to CDSS implementation. It addressed key BEAR domains; human factors, clinical context, and implementation by assessing nurses’ perceptions, intravenous drug administration practices, as well as specific feedback on the Swedish Y-site compatibility system. However, the framework was used to support questionnaire development rather than as a predefined analytical framework.

The digital survey was created in REDCap version 14.0.12 (© 2024 Vanderbilt University) and distributed in 2023 to all 296 eligible intensive care unit nurses via workplace email. It was organized into four sections: participant demographics, intravenous drug administration practices, general perceptions of clinical decision support systems, and perceptions of the Swedish Y-site compatibility system. Questions and statements were formatted as either a four-level Likert scale with an additional “Cannot or will not answer” option, or as open-ended or Yes/No questions.

### 2.4 Data analysis

Descriptive statistics were calculated for demographic variables and survey responses using Microsoft® Excel® for Microsoft 365 MSO (Version 2408). As responses were collected using an ordinal Likert scale, items endorsed by ≥60% of respondents were classified a priori as key barriers or facilitators, representing a clear majority of respondents. Missing responses for individual survey questions were handled by calculating percentages based on the number of respondents who answered that specific question, rather than the total sample size.

### 2.5 Ethics

The Swedish Ethical Review Authority evaluated the study and determined it was not subject to ethical review, as it did not include any elements requiring ethical approval under Swedish law (Dnr 2022–07,289-01).

## 3 Results

### 3.1 Respondents of the survey

A total of 126 intensive care unit nurses (43%) responded to the survey. Mostly female (85%, n = 107) and 54% had >10 years of intensive care unit experience. Respondents worked in a variety of intensive care units, with the highest proportion working in the general intensive care unit (55%, n = 69) (Table 1).

**Table 1:** Respondents’ demographic characteristics.

Demographic	N (%)
Respondents	126 (43)
Age (years)	
≤29	10 (8))
30–39	32 (25)
40–49	28 (22)
50–59	34 (27)
≥60	21 (17)
Missing value	1 (1)
Gender	
Women	107 (85)
Man	17 (13)
Missing value	1 (1)
Years of ICU* experience	
0–1	14 (11)
2–5	18 (14)
5–10	25 (20)
>10	68 (54)
Missing value	1 (1)
Workplace (ICU*)	
General	69 (55)
Neurological	11 (9)
Thorax	45 (36)
Missing value	1 (1)

Abbreviation; *Intensive Care Unit.

### 3.2 Intravenous drug administration

Out of 126 respondents, nearly all (n = 122, 97%) were actively involved in administering intravenous medication, the remaining four (3%) were registered as “Missing value”. Among the 122 who reportedly administered intravenous medications as part of their work, a Majority of 67% (n = 84) reported having administered intravenous medication at some point without knowing the risk of potential intravenous incompatibility. Additionally, those who had administered intravenous medication without knowing its Y-site compatibility potential (n = 84), answered that they encounter this either daily (n = 4, 5%), once a week (n = 11, 13%), once a month (n = 22, 26%), or less than once a month (n = 47, 56%).

**Fig. 1:**
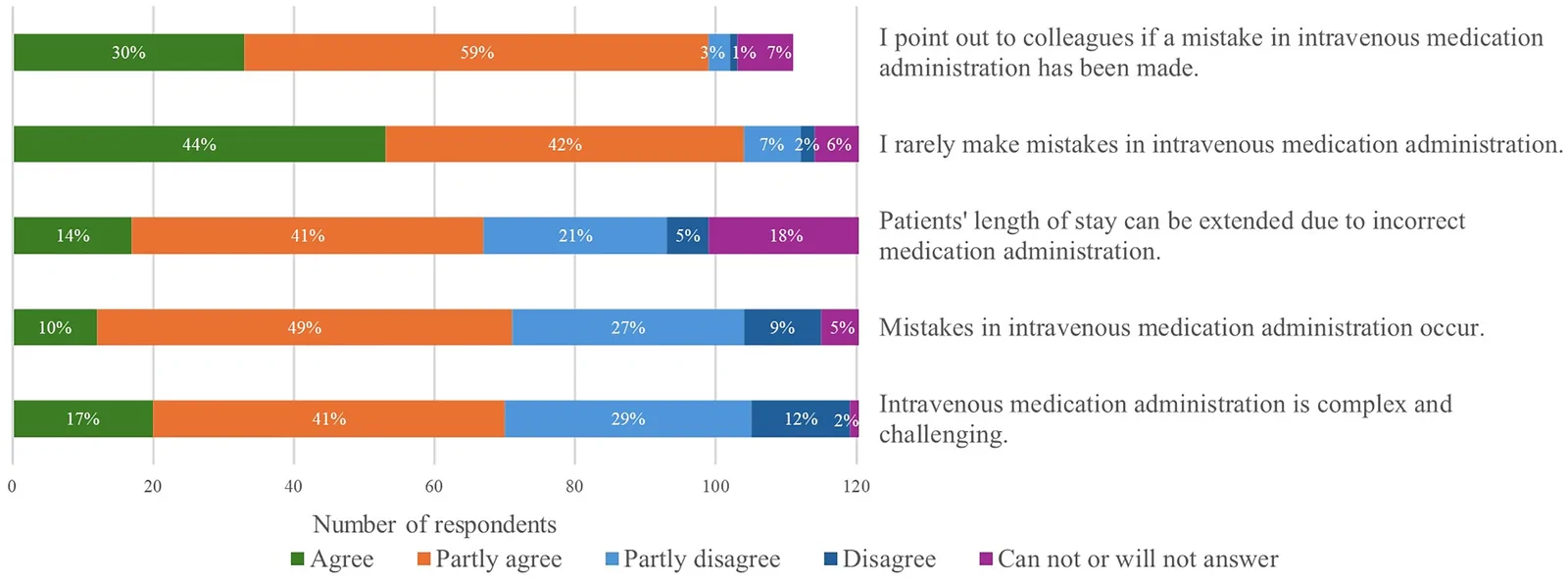
Questions and responses related to intravenous medication errors and their perceived complexity. The X-axis displays the response counts, which are represented by the bars. The percentages of respondents are shown on the bars.

Many of the 121 respondents recognized the complexity of intravenous drug administration, with more than half (n = 70, 58%) describing it as challenging (Fig. 1). Despite this, most participants felt confident in their own practice, reporting that they rarely make mistakes during administration (n = 104, 86%). At the same time, a majority (n = 71, 59%) acknowledged that errors do occur in general, and the Majority (n = 99, 82%) actively provide feedback to colleagues when mistakes are identified. Additionally, 55% (n = 67) of the respondents state that, in their opinion, medication errors could be associated with prolonged hospital stays for patients. When asked about errors among peers, nearly all respondents and (n = 99, 89%) indicated that they point out mistakes in intravenous medication administration, reflecting a culture of vigilance and mutual accountability.

### 3.3 Sweden’s Y-site compatibility database

**Fig. 2:**
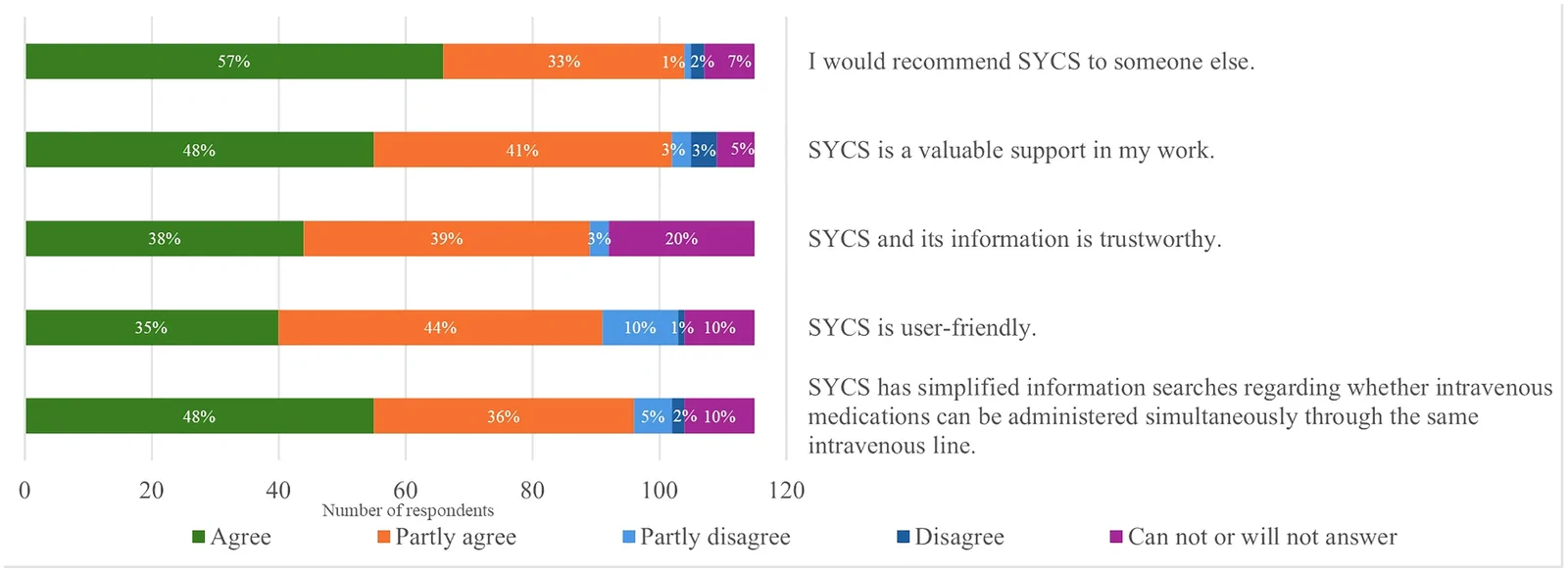
Questions and responses regarding the use and perceptions of the Swedish Y-site compatibility system (SYCS). Counts are displayed along the X-axis, and on the bars the percentage of respondents is illustrated.

A total of 115 respondents answered the questions regarding the Swedish Y-site compatibility system (Fig. 2), and 11 (9%) were recorded as missing value. A majority, (n = 102, 89%) were using the Swedish Y-site compatibility system, and 13 (11%) respondents reported they were not using it. The open-end question for those not using the Swedish Y-site compatibility system highlighted barriers such as limited awareness, accessibility, and practicality, limited time to use clinical decision support systems, digital inaccessibility, and insufficient resources (e.g., intravenous access) for it to be useful. There was also concern regarding the database's update frequency. The only other source of information on intravenous medication compatibility mentioned by respondents was a locally used printed chart.

Overall, respondents expressed a highly positive attitude towards the Swedish Y-site compatibility system, with most indicating that they would recommend it to others (n = 104, 90%). A majority, 89% (n = 102) responded positively about the value of the Swedish Y-site compatibility system to their work; for instance, 83% (n = 96) agreed to some extent that the Swedish Y-site compatibility system has improved their ability to find information regarding Y-site compatibility. A majority (n = 91, 79%) agreed to some extent that the Swedish Y-site compatibility system is user-friendly and trustworthy (n = 89, 77%).

The question on desired functionalities in the Swedish Y-site compatibility system was presented as a multiple-choice item allowing multiple selections, accompanied by an open-ended response option. A total of 123 respondents answered the question with at least one functionality selection. The most frequently requested functionality was the ability to generate printable compatibility overviews (n = 85, 69%). Second, the guidance on appropriate intravenous administration based on intravenous access type (central vs. peripheral) (n = 67, 69%) and reconstitution instructions for intravenous drugs (n = 67, 54%), information on co-administration suitability based on pharmacological effects (n = 35, 28%). Moreover, 9 respondents expressed interest in the possibility of requesting medication combinations for intravenous drug compatibility assessment to be put in the Swedish Y-site compatibility system. Seven respondents selected "Other" and provided open-ended feedback, highlighting the need for clarification on contraindications for co-administration, such as risks of bolus dosing or precipitation, and guidance on peripheral administration suitability. They also emphasized the importance of including comprehensive data on commonly used intravenous drugs in intensive care units, especially where evidence is limited, and one respondent called for further investigation into drug potentiation effects when multiple agents are combined.

An open-ended question about which sources are used for intravenous co-administration had 104 respondents and a total of 201 identified sources for information. The following sources for information about Y-site compatibility was listed; Summary of Product Characteristics (FASS [Internet], 2018) (n = 74, 71%), guideline (n = 45, 43%), the Swedish Y-site compatibility system (n = 41, 39%), colleague (presumed intensive care unit nurse colleague; n = 19, 18%), other clinical decision support systems (n = 6, 6%), clinical pharmacist (n = 6, 2%), physician (n = 2, 2%), own experience (n = 2, 2%) and other sources (n = 5, 5%), such as information from other regions in Sweden or printer systems for medication labels.

### 3.4 Barriers and facilitators for clinical decision support systems in the intensive care unit

An apparent facilitator was that a vast Majority (n = 99, 89%) of respondents responded that clinical decision support systems improve medication administration and 92% believed it is valuable for patient care. Clinical decision support systems were not perceived as affecting clinical autonomy and independence.

No social factors in the workplace proved to be a relevant barrier. Most respondents (72%) indicated some level of disagreement with the statement suggesting a negative attitude toward software support in the intensive care unit, implying generally positive perceptions of such support. No clear influence of hierarchy was detected opinions of senior colleagues were not relevant to most respondents. Intriguingly, one-quarter of participants (n = 27, 25%) emphasized the importance of colleague competence in the intensive care unit. Indicating there is a culture of working together as a team, rather than as senior and junior colleagues, to solve medication related issues.

**Fig. 3:**
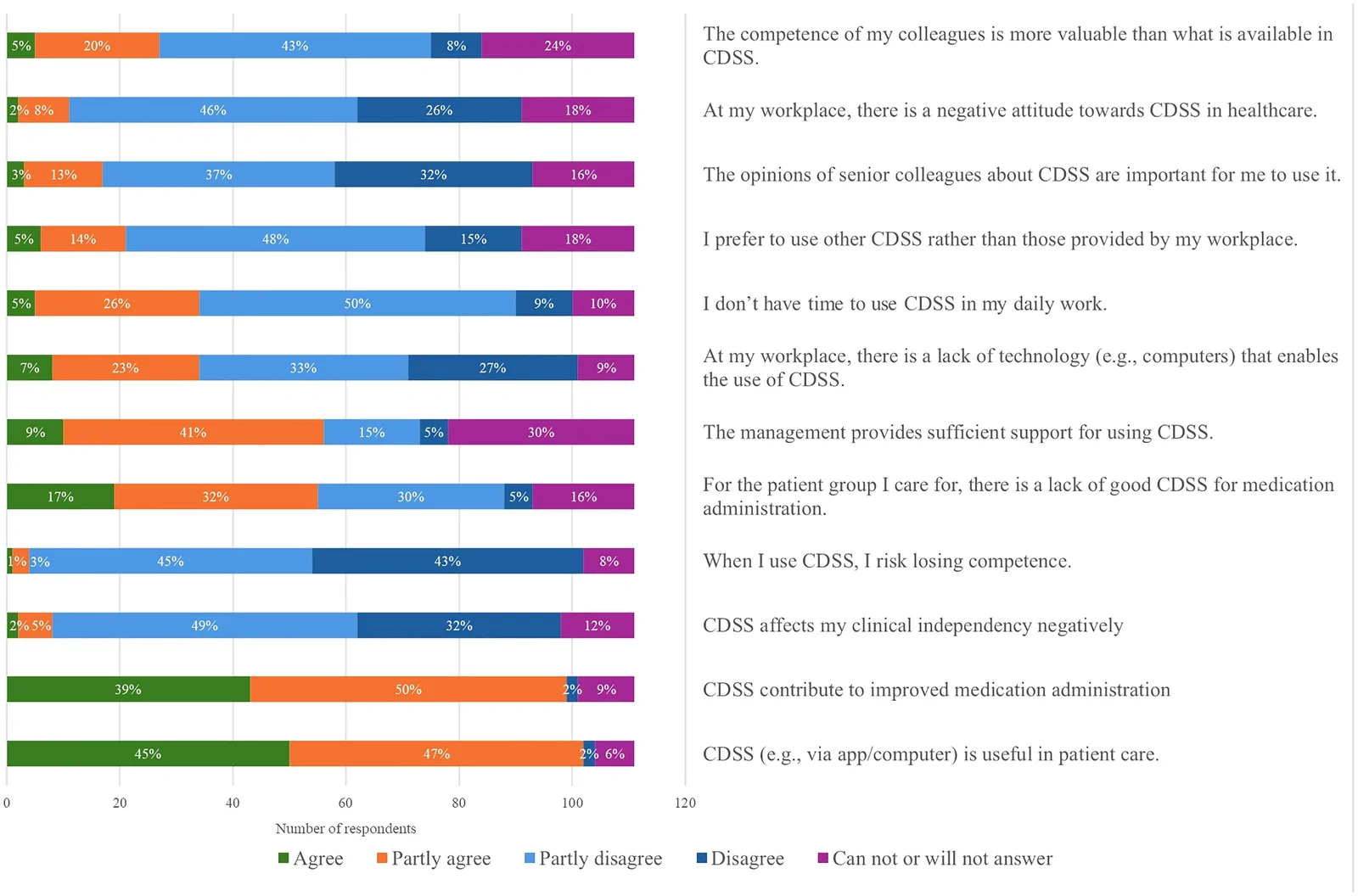
Questions and responses concerning clinical decision support systems (CDSS) in the intensive care unit setting. The X-axis and bars show counts, and the percentage of respondents is indicated on the bars.

The potential barrier regarding lack of time to utilize clinical decision support systems did not appear to be a significant obstacle, as 59% of respondents disagreed. However, 31% (n = 34) did agree or party agree to the statement of not having enough time to use a clinical decision support system in their clinical work. Similarly, perceived lack of access to the necessary technology elicited comparable levels of disagreement (n = 67, 60%). This was suggesting that resource availability may not be a major barrier, but 31% (n = 34) of respondents still considered it an issue. Regarding organizational support, 45% of respondents (n = 56) stated that management provided adequate support for clinical decision support system use. Across all items presented in Fig. 3, the rate of missing responses was 12% (n = 15), highlighting a modest level of nonresponse. A total of 49% (n = 55) of respondents agreed och party agreed to the statement of there not being a good clinical decision support system for their patient group.

## 4 Discussion

### 4.1 Intravenous drug administration

This survey addresses clinical decision support system used in the intensive care unit regarding use and Y-site drug compatibility—an area linked to serious clinical consequences, including line occlusion, emboli, and even mortality (Benlabed et al., 2019; Bradley et al., 2026). Nearly all respondents (97%) were actively involved in intravenous drug administration, and 18% reported weekly uncertainty about drug compatibility. This indicates gaps in available knowledge or evidence, or that existing resources such as the Swedish Y-site compatibility system may not fully meet the needs of intensive care unit practice.

Previous work by Kanji et al. (2013) and Taxis (2003), Taxis and Barber (2004), together with our findings, show that co-administration challenges are routine practices in the intensive care unit. The frequent incompatibility issues reported here, combined with the fact that more than half of commonly co-administered drug pairs in intensive care lack published compatibility data (Kanji et al., 2010; D’Huart et al., 2019; Castells Lao et al., 2020), underscore the difficulty of ensuring safe intravenous medication administration and the substantial evidence gaps clinicians must navigate.

Although most respondents reported rarely making mistakes (86%), the high recognition of medication errors (59%) and willingness to point them out suggest a strong safety culture among intensive care unit nurses. This indicates that even when individuals feel confident in their practice, they remain vigilant and engaged in maintaining patient safety. Over half of respondents (55%) believed that medication errors contribute to prolonged hospital stays, reflecting awareness of their clinical impact. However, the uncertainty expressed by some (18%) highlights a need for further education on medication-error prevention, adverse drug events, and medication safety—especially as research has established a clear association between medication errors and increased intensive care unit length of stay (Classen et al., 1997; Vargas et al., 1998; Nuckols et al., 2008; Vargas et al., 2003).

### 4.2 Sweden’s Y-site compatibility database

The findings suggest that the Swedish Y-site compatibility system holds an important role in intensive care unit medication practice, functioning as the primary resource for intravenous Y-site compatibility among respondents. The absence of international references such as UpToDate, LexiDrug (UpToDate Lexidrug 2025) or Micromedex (MICROMEDEX [Internet] 2025) in the open-ended responses indicates that these databases may not be integrated into local workflows or perceived as sufficiently tailored to Swedish intensive care unit contexts.

Although the Swedish Y-site compatibility system was specifically designed to provide information on intravenous drug compatibility, FASS (2018) was reported as a frequently consulted source among respondents (71%). One possible explanation is that FASS is a well-established and familiar medication information source in Swedish healthcare, providing access to the Summary of Product Characteristics and other medication-related information routinely used by healthcare professionals. However, FASS generally does not provide information regarding drug–drug compatibility or Y-site compatibility, meaning that it cannot replace dedicated compatibility resources such as the Swedish Y-site compatibility system for these specific clinical decisions. The continued preference for FASS may therefore reflect established routines and familiarity with existing information sources rather than a preference for compatibility information provided by FASS. These findings highlight that availability of a specialized clinical decision support system alone may not be sufficient to ensure its integration into clinical practice, and that implementation strategies supporting routine use in relevant clinical situations may be needed.

As many as 67% reported administering intravenous medications without knowing their compatibility. At first glance, these findings may appear contradictory. However, access to or general use of a compatibility resource, such as the Swedish Y-site compatibility system, does not necessarily equate to its consistent use at the point of care. In the complex and time-critical intensive care environment, clinicians may encounter situations in which compatibility information is not readily available, cannot be located quickly enough, or the resource is not consulted because of workflow demands. Furthermore, this finding may also reflect limitations in the current evidence base for intravenous drug compatibility. Compatibility data are lacking for many drug combinations, and pharmaceutical manufacturers rarely provide comprehensive compatibility information for co-administered intravenous medications. Consequently, even when nurses consult available resources, definitive compatibility information may not exist. These findings suggest that current resources, while widely used, do not always meet clinicians' information needs during medication administration and highlight the need for more comprehensive, accessible, and workflow-integrated clinical decision support.

Overall, respondents viewed the Swedish Y-site compatibility system positively, yet the notable proportion expressing uncertainty points to the possibility of improvement. Although 89% of respondents reported using Sweden’s Y-site compatibility system, nearly half of the respondents (49%) agreed or partly agreed that there is no adequate clinical decision support system available for their patient group. These findings suggest that, while existing resources provide valuable support for specific tasks such as intravenous drug compatibility assessment, they may not fully address the broader medication-related decision-making needs of intensive care nurses. This indicates that there may be room for improvement and further development of clinical decision support systems tailored to the intensive care context. However, the specific functionalities, design, and implementation approach required to best support clinicians remain unclear and require further investigation. Future research should therefore explore clinicians’ needs, workflows, and expectations to guide the development of clinically relevant and user-centred decision support solutions. This aligns with previous research highlighting persistent gaps in medication-support systems within intensive care unit settings, including the work of Bakker et al. (2024), which similarly identified the need for enhanced decision-support tools. Taken together, these observations indicate that while the Swedish Y-site compatibility system is a trusted and widely used resource, opportunities remain to refine and expand its support to better match the complexities of intensive care unit medication management.

### 4.3 Barriers and facilitators for clinical decision support systems in the intensive care unit

While no single barrier reached the ≥60% threshold, the results still point to a clear need for a whole-system approach to safer intravenous medication administration in the intensive care unit. Items endorsed by ≥60% of respondents were classified a priori as key barriers or facilitators. Predefined percent agreement thresholds are commonly used in survey and consensus research, with reported cutoffs ranging from 50% to 80% depending on study context (Diamond et al., 2014). None of the proposed barriers such as time constraints, or threats to clinical autonomy and integrity had ≥60% response rate (Fig. 2). In contrast to Liberati et al. (2017) where health care personnel viewed clinical decision support systems as a threat to their clinical autonomy, this study did not find this barrier among intensive care unit nurses (Liberati et al., 2017).

This survey reveals generally positive perceptions of clinical decision support systems as a facilitator in clinical decision support system use in the intensive care unit setting. This might be due to Sweden’s high level of digitalization (Sweden in the Digital Economy and Society Index | Shaping Europe’s digital future [Internet] 2025), and the widespread usage of nationally coordinated clinical decision support systems, for example “Svensk informationstjänst för läkemedel” (Sil) (Sil Online [Internet] 2025), Janusmed (Janusmed [Internet] 2025) and ePed (ePed [Internet] 2025). Nevertheless, it is important to acknowledge that such a positive attitude may not be uniformly reflected across all intensive care unit settings globally.

Positive attitudes toward clinical decision support systems suggest a supportive culture for digital decision aids, but culture alone does not ensure consistent use at the bedside—especially in high-acuity situations where workarounds and “good enough” decisions may emerge under pressure. To translate acceptance into safer practice, structures must enable clinical decision support systems to be used as a natural part of workflow (e.g., easy access at the point of preparation/administration, alignment with intensive care unit infusion practices, and clear local routines for compatibility checks). Finally, training is needed to strengthen competence in compatibility risk assessment and to normalize clinical decision support systems-supported decision-making, particularly given the substantial proportion of nurses who administer intravenous drugs without confirmed compatibility. Together, a reinforcing combination of culture, structure, and training is required to embed clinical decision support systems as a reliable safety net rather than a tool used only when time permits.

### 4.4 Further development of a clinical decision support system

To the authors' knowledge, there is currently no clinical decision support system specifically tailored to intravenous medication management in the intensive care unit that addresses intravenous Y-site drug compatibility or specific intensive care unit related drug information. According to previous attempts, there is room for improvement in drug-related outcomes if a clinical decision support system is developed specifically for the intensive care unit. For example, a study designed to improve drug-drug interaction management using a customized clinical decision support system showed positive results in clinical outcomes; less potentially harmful interactions and patient length-of-stays in this setting (Bakker et al., 2024).

The question regarding desired functionality in the Swedish Y-site compatibility system did show room for improvement, as most respondents requested several additional features. Furthermore, 55 (47%) respondents did express that there is a lack of clinical decision support system for drug administration and information for their specific patient group. These findings align with previous research suggesting that technical and contextual factors are essential for maximizing effectiveness of clinical decision support systems (Amaral and Cuthbertson, 2024; Oduyale et al., 2020; Trivedi et al., 2002). This finding aligns with a previous study by Bakker T et al. that have highlighted the importance of dedicated and tailored clinical decision support systems adapted to the intensive care unit context (Bakker et al., 2024), and particularly regarding Y-site drug compatibility in particular according to intensive care unit nurses (Oduyale et al., 2020). According to Jansson et al., there are gaps in design, usability, and content reliability regarding clinical decision support systems used in the intensive care unit, and that needs to be further studied and addressed in development of such a tool (Jansson et al., 2022).

### 4.5 Study limitations

Survey-based research carries inherent limitations, yet it remains useful for capturing clinicians’ perspectives. In this study, the overall response rate of 43%, together with lower completion rates for some items, limits generalizability. Similar studies in comparable settings have reported even lower participation, but still yielded meaningful conclusions (Wong et al., 2023). Partially completed surveys add to these limitations and are a common challenge in digital data collection among healthcare workers, whose workflows are frequently interrupted. Both non-response and incomplete data introduce potential selection bias and further constrain the transferability of findings.

As this study was conducted at a single Swedish university hospital, the findings may be influenced by local factors, including implementation processes, user training, and organizational support for the CDSS. Although the system is available for use throughout Sweden, it was originally developed and implemented within the regional healthcare organisation where the university hospital in this study is located. This may have resulted in greater familiarity, experience, and integration of the system among users in this study. Therefore, the positive attitudes observed may partly reflect local implementation quality rather than general ICU culture. This may limit the transferability of the findings, as nurses working in other healthcare organisations may have different levels of exposure to and experience with the system. Multicentre studies, including sites with varying levels of CDSS experience are needed to further assess the generalizability of these findings.

Although the questionnaire was not analysed according to the BEAR framework, the findings align with several of its domains, particularly those related to workflow integration, user needs, and system characteristics. No clear barriers or facilitators were identified based on our predefined threshold of ≥60% agreement. However, a substantial proportion of respondents (30–49%) expressed concerns within these domains, indicating areas that may warrant further attention during future CDSS development and implementation. These findings should be interpreted cautiously, as the use of a predefined agreement threshold in a study with a modest sample size may reflect the perspectives of only a limited number of respondents and may not fully represent the broader intensive care nurse population. Nevertheless, the concerns identified by a considerable minority of respondents provide valuable insights into potential challenges that should be explored further, particularly through qualitative research and clinician involvement in future co-creation processes.

### 4.6 Future research

This survey has provided a useful starting point for understanding intensive care unit nurses' needs in relation to a clinical decision support system for medication administration. The findings provide a foundation for further investigation using qualitative methods, such as semi-structured interviews, to explore clinical workflows, usability requirements, and functional expectations in greater depth. In subsequent development phases, a co-creation approach involving intensive care clinicians could be adopted to ensure that end users actively contribute to the design process. Engaging clinicians throughout development may help ensure that the system reflects clinical practice, addresses user needs, and facilitates acceptance and implementation in routine care.

## 5 Conclusion

This study underscores the role of clinical decision support systems as practical decision aids for intravenous drug compatibility in the intensive care units, where time pressure, complex multi-infusion regimens, and rapid clinical changes make compatibility decisions difficult to manage from memory alone. intensive care unit nurses largely perceived an evidenced based Y-site drug compatibility clinical decision support system as valuable support for safer intravenous medication administration and were willing to recommend it. However, the finding that many nurses still administer intravenous medications without knowing compatibility indicates persistent vulnerability in everyday practice and suggests that decision aids are not consistently integrated at the point of care. Key challenges include ensuring easy, rapid access during urgent situations, aligning the system’s content and workflow with intensive care unit -specific infusion practices, and reinforcing use through targeted education and organizational support. Further development and implementation strategies may strengthen the Swedish Y-site compatibility system as a real-time compatibility decision aid and reduce avoidable medication risks in the intensive care unit setting.

## Funding source information

This research did not receive any specific grant from funding agencies in the public, commercial, or not-for-profit sectors.

## CRediT authorship contribution statement

**Annabel Peyravi Latif:** Writing – review & editing, Writing – original draft, Visualization, Validation, Software, Project administration, Methodology, Investigation, Formal analysis, Data curation, Conceptualization. **Alessio Degl’Innocenti:** Writing – review & editing, Validation, Supervision, Resources, Methodology, Funding acquisition, Conceptualization. **Bengt Nellgård:** Writing – review & editing, Validation, Supervision, Resources, Methodology, Conceptualization. **Axel Wolf:** Writing – review & editing, Validation, Supervision, Resources, Methodology, Investigation, Funding acquisition, Formal analysis, Conceptualization.

## Declaration of competing interest

The authors declare the following financial interests/personal relationships which may be considered as potential competing interests: Bengt Nellgard reports administrative support and statistical analysis were provided by University of Gothenburg. Bengt Nellgard reports a relationship with University of Gothenburg Sahlgrenska Academy that includes: employment. None If there are other authors, they declare that they have no known competing financial interests or personal relationships that could have appeared to influence the work reported in this paper.

## Appendix Supplementary materials

Image, application 1
