# Supplementary material for: Barriers and facilitators for intensive care nurses in using clinical decision support systems for intravenous Y-site compatibility in intensive care: A survey study
Source: Int J Nurs Stud Adv. 2026 Jul 20;11:100637. doi: 10.1016/j.ijnsa.2026.100637 (PMC13542926; doi:10.1016/j.ijnsa.2026.100637)
Supplement: Supplementary file 1 [file mmc1.pdf]

BESLUT OCH YTTRANDE

2023-02-21

Sökande forskningshuvudman
Göteborgs universitet

Forskare som genomför projektet
Bengt Magnus Gustaf Nellgård

Projekttitel
Mixed methods studier av mjukvarustöd för intravenös läkemedelsadministrering

Uppgifter om ansökan
Ansökan inkom till Etikprövningsmyndigheten 2022-12-28 och blev valid 2023-01-23.

Etikprövningsmyndigheten beslutar enligt nedan. Etikprövningsmyndigheten lämnar samtidigt ett rådgivande yttrande enligt 4 a § förordningen (2003:615) om etikprövning av forskning som avser människor.

Etikprövningsmyndigheten
2022-07289-01-378705
2023-03-07

BESLUT

Etikprövningsmyndigheten avvisar ansökan, det vill säga tar inte upp ansökan till prövning.

Skäl för beslutet
I det aktuella projektet kommer det inte att göras något ingrepp på en forskningsperson eller annan intervention på sätt som anges i 4 § etikprövningslagen. Det kommer inte att ske någon behandling av personuppgifter på så sätt som anges i 3 § etikprövningslagen. Mot bakgrund härav omfattas inte studien av bestämmelserna i 3-4 §§ etikprövningslagen och ska därför inte etikprövas.

RÅDGIVANDE YTTRANDE

Etikprövningsmyndigheten har inte några etiska invändningar mot den beskrivna enkätdelen av projektet.

Framtida forskning, den uppföljande intervjustudien, måste etikprövas om känsliga personuppgifter hanteras.

Beslutet att inte ta upp ansökan till prövning kan överklagas hos Överklagandenämnden för etikprövning. Hur man överklagar framgår av bifogad anvisning. Det rådgivande yttrandet får inte överklagas.

På Etikprövningsmyndighetens vägnar

Hanna Werth
Ordförande

Beslutet har fattats av följande personer:

Ordförande
Hanna Werth (Rådman)

Ledamöter med vetenskaplig kompetens
Oonagh Shannon (Mikrobiologi, Immunologi, vetenskaplig sekreterare)
Kristina Källén (Neurologi, föredragande)
Emily Sonestedt (Nutrition, epidemiologi, genetik, kardiometabola sjukdomar)
Eva Brun (Onkologi)
EwaCarin Ekberg (Odontologi)
Daniel Förnvik (Sjukhusfysiker)
Maria Landqvist Waldö (Psykiatri)
Pouya Movahed (Psykiatri, experimentell farmakologi, smärta)
Carl Johan Tiderius (Ortopedi, Barnortopedi, Artros)

Ledamöter som företräder allmänna intressen
Jerry Bergström
Cristina Glad
Lars Karlsson

Beslutet sänds till
Ansvarig forskare: Bengt Magnus Gustaf Nellgård
Forskningshuvudmannens företrädare: Jovanna Dahlgren

Etikprövningsmyndigheten
2022-07289-01-378705
2023-03-07

Hur man överklagar Etikprövningsmyndighetens beslut

Vem får överklaga?

Det är forskningshuvudmannen som får överklaga Etikprövningsmyndighetens beslut om det har gått sökanden emot. Överklagandet ska vara skriftligt. Skrivelsen ska vara undertecknad av behörig företrädare för forskningshuvudmannen.

Om forskaren överklagar ska en fullmakt från forskningshuvudmannen bifogas.

När ska beslutet senast överklagas?

Överklagandet ska ha kommit in till Etikprövningsmyndigheten inom tre veckor från den dag då forskningshuvudmannen fick del av beslutet.

Vad ska överklagandet innehålla?

Överklagandet ska innehålla uppgifter om

1. klagandens namn, person- eller organisationsnummer, adress, telefonnummer och e-postadress
2. det beslut som överklagas (dag för beslut, projekttitel och diarienummer)
3. hur ni anser att myndighetens beslut ska ändras och skälen till att beslutet bör ändras.

Var ska överklagandet skickas?

Överklagandet ska ställas till Överklagandenämnden för etikprövning. Men det ska skickas eller lämnas till Etikprövningsmyndigheten.

Om överklagandet har kommit in i rätt tid överlämnar myndigheten överklagandet och handlingarna till Överklagandenämnden för etikprövning.

Etikprövningsmyndigheten
2022-07289-01-378705
2023-03-07

registrator@etikprovning.se | 010-475 08 00 | Box 2110, 750 02 Uppsala | etikprovning.se
